# Digital CXR with computer aided diagnosis versus symptom screen to define presumptive tuberculosis among household contacts and impact on tuberculosis diagnosis

**DOI:** 10.1186/s12879-017-2388-7

**Published:** 2017-04-24

**Authors:** Monde Muyoyeta, Nkatya Chanda Kasese, Deborah Milimo, Isaac Mushanga, Mapopa Ndhlovu, Nathan Kapata, Maureen Moyo-Chilufya, Helen Ayles

**Affiliations:** 1 ZAMBART Project, University of Zambia, School of Medicine, Lusaka, Zambia; 2 National TB program, Ministry of Health, Lusaka, Zambia; 3 Clinical research Department, Faculty of Infectious and Tropical Diseases, London School of Hygiene and Tropical Medicine, London, UK

**Keywords:** Tuberculosis, Digital CXR, CAD, Household contact, Diagnosis, Screening, IPT, Zambia

## Abstract

**Background:**

Household (HH) contact tracing is a strategy that targets high risk groups for TB. Symptom based screening is the standard used to identify HH contacts at risk for TB during HH contact tracing for TB. However, this strategy may be limited due to poor performance in predicting TB. The objective of this study was to compare CXR with Computer Aided Diagnosis (CAD) against symptom screen for defining presumptive TB and how TB detection changes with each method.

**Methods:**

Household contacts of consecutive index bacteriologically confirmed TB cases were visited by study teams and given TB/HIV education to raise awareness of the risk of TB following close contact with a TB patient. Contacts were encouraged to visit the health facility for screening; where symptoms history was obtained and opt out HIV testing was provided as part of the screening process. CXR was offered to all regardless of symptoms, followed by definitive sputum test with either Xpert MTB RIF or smear microscopy.

**Results:**

Among 919 HH contacts that presented for screening, 865 were screened with CXR and 464 (53.6%) had an abnormal CXR and the rest had a normal CXR. Among 444 HH contacts with valid sputum results, 274 (61.7%) were symptom screen positive and 255 (57.4%) had an abnormal CXR. Overall, TB was diagnosed in 32/444 (7.2%); 13 bacteriologically unconfirmed and 19 bacteriologically confirmed. Of 19 bacteriologically confirmed TB 8 (42.1%) were symptom screen negative contacts with an abnormal CXR and these 6/8 (75.0%) were HIV positive. Among the 13 bacteriologically unconfirmed TB cases, 7 (53.8%) were HIV positive and all had an abnormal CXR.

**Conclusion:**

Symptom screen if used alone with follow on definitive TB testing only for symptom screen positive individuals would have missed eight of the 19 confirmed TB cases detected in this study. There is need to consider use of other screening strategies apart from symptom screen alone for optimal rule out of TB especially in HIV positive individuals that are at greatest risk of TB and present atypically.

**Electronic supplementary material:**

The online version of this article (doi:10.1186/s12879-017-2388-7) contains supplementary material, which is available to authorized users.

## Background

Household (HH) contacts of newly diagnosed tuberculosis (TB) patients are considered to be a high risk group for TB [1–3]. In view of this, household contact tracing is recommended as one of the strategies for TB control. The World Health Organisation (WHO) recommends that HH contacts that have symptoms suggestive of presumptive TB, People Living with HIV (PLHIV) and children under 5 years old must undergo clinical evaluation for TB [4]. Typically, among HIV positive individuals, a person is considered to be a presumptive TB patient if they have any one of the symptoms; cough, fever, night sweats or weight loss [5, 6]. Among HIV negative patients, a person is generally considered to be a presumptive TB patient if they have a cough greater or equal 2 weeks duration with or without other symptoms of TB. Symptom screen to define presumptive TB has been observed to result in missed active TB cases among those who are symptom screen negative and are not considered for follow on testing with definitive TB tests [7–9].

In 2016, the WHO recommendations for TB screening included Chest X-ray (CXR) as a screening tool that is more sensitive than symptom screen [10]. This is in recognition of the potential role of CXR in TB diagnosis especially with newer digital chest x-ray technology and advances to electronically read CXRs making it possible for CXR to be readily available in resource constrained settings with high burden of TB and HIV and scarce personnel to interpret CXRs [11–14]. New advances in TB diagnostics such as Xpert MTB/RIF may also require adjunct pre-screening tests that are highly sensitive and have high negative predictive value, that reduce the number needed to screen to detect one case of TB, ultimately reducing the cost of TB diagnosis [15–17].

HH contact tracing for contacts of patients with bacteriologically proven TB was conducted as part of an implementation evaluation study of Xpert MTB/RIF, used in conjunction with digital CXR with Computer Aided Diagnosis (CAD) [18, 19]. In this study, we evaluated using CAD as a tool to define presumptive TB as opposed to the traditional method that uses symptom screening among HH contacts of newly diagnosed TB patients with bacteriologically confirmed TB.

### Ethical considerat**i**ons

The study had ethical approval from the University of Zambia Biomedical Research Ethics Committee. Permission to visit the household was initially obtained from the TB patient and written informed consent was obtained from the head of the household.

## Methods

### Study population and study setting

The study population comprised all household contacts of patients diagnosed with bacteriologically confirmed TB. This study was conducted in one government health facility in Lusaka, Zambia.

### Procedures

Newly diagnosed TB patients were informed about the importance of screening household (HH) members for TB. In the household, an enumeration form was completed and this form collected data on the total number of HH members disaggregated by age and sex, the total number of rooms in the house and the total number of rooms used for sleeping in.

TB and HIV information/education was given to those present at the time of the visit, aimed at raising awareness of the risk of TB following close contact exposure, the increased risk for HIV positive individuals to develop TB following exposure and why it was important to be screened for both HIV and TB. All screening for TB and HIV were done from the health facility and HH members were invited to visit the health facility for screening.

**Fig. 1:**
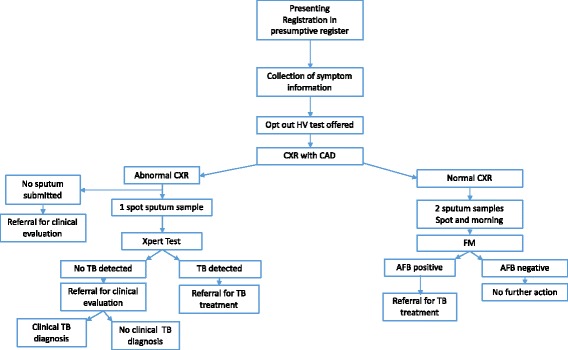
Diagnostic Algorithm

At the health facility, all presenting HH contacts were registered in the presumptive TB register and allocated a unique identification number. The index identification number was also recorded for purposes of linking the HH contact to the index patient. All HH contacts were offered HIV counselling and opt out HIV testing if they did not know their HIV status. CXR was also offered to all HH members and further testing of sputum with Xpert was offered to those with an abnormal CXR whilst those with a normal CXR were offered smear microscopy according to the algorithm being evaluated (Fig. 1). The full description of the algorithms being tested has been given elsewhere [18, 19].

Briefly, using CAD (CAD4TB, version 1.08, Diagnostic Image Analysis Group, Nijmegen, the Netherlands) [14, 20] to electronically read CXRs, with set thresholds defining normal CXR (CAD less than 61) and abnormal CXR (CAD score greater than or equal to 61), HH members with an abnormal CXR who were able to give sputum were tested with Xpert, whilst those with normal CXR were tested using smear microscopy according to the algorithm that was being tested. Procedures for Xpert testing and smear microscopy have been described elsewhere and followed standard operating procedures [18, 19].

HH members with newly diagnosed HIV infection were referred for HIV care under the routine services. Children not diagnosed with TB were referred for IPT under routine services according to the existing national guidelines at the time. And all patients with TB detected were referred to start TB treatment. Patients with no TB detected had clinical evaluation and appropriate management given as determined by the attending clinician. Those commenced on TB treatment based on clinician’s judgement were defined as having a clinical TB diagnosis.

### Data collection

The data presented in this study was collected between May 2013 and March 2014. Data collected on paper based HH enumeration forms was double entered in an access data base. Using the index barcode as the unique identifier, HH enumeration data was merged with presumptive TB register data and TB treatment register. For presumptive TB registration, an electronic register was used which was based on routine presumptive TB registers in use. The database was designed and developed using Microsoft Structured Query Language (SQL) Server 2005 as the backend and Microsoft Office Visual Basic.Net as the front end (Microsoft Corporation). Validation checks were also created to ensure that data were entered correctly. To ensure the correctness of data, data entry standards were developed on how to correctly capture data and how to handle missing data.

### Data analysis

Data analysis was conducted using STATA (Stata Corporation Version 13. College Station, TX, USA). Initial descriptive analysis was conducted to show the number of index TB cases accepting to have HH contact tracing as well as to show the total number of household members that were enumerated disaggregated by age (Fig. 1.) Further descriptive analysis was conducted to show the median HH size, and median number per sleeping room (Table 1). Baseline characteristics of HH contacts that presented for screening are shown in Table 2. To understand the impact of CAD on TB detection in relation to symptom screening, analysis was conducted disaggregated by HIV status to take care of the different definitions of positive symptom screen (presumptive TB) among HIV positive and HIV negative individuals (Figs. 3 and 4). Contacts with unknown HIV status were grouped with HIV positive individuals. Among HIV positive/unknown status HH contacts, they were considered to have a positive symptom screen if they had any of cough, fever, night sweats or weight loss. Among HIV negative contacts, symptom disaggregation was; no cough present, cough less than 2 weeks and cough for 2 weeks or more with or without other symptoms.

**Table 1:** Household characteristics (Total number enumerated in HH visited 4297)

Total Enumerated	4297(%)
Distribution by age	
Total adults > = 15 years	2655 (61.8%)
Total 5–14 years	928 (21.6%)
Total < 5 years	714 (16.6%)
Median number of people/HH (IQR)	4 (3–6)
Median number rooms/HH (IQR)	2 (2–3)
Median number of sleeping rooms (IQR)	2 (1–2)
Median number of people/sleeping room (IQR)	2 (2–3)

**Table 2:** Baseline characteristics of Household contacts presenting to the health facility

Characteristic	*N* = 919 (%)
Median age (IQR)	15 (6–31)
Female Sex	549 (59.7)
Cough and duration	
No cough	663 (72.1)
< 2 weeks	172 (18.7)
2–8 weeks	67 (7.3)
>8 weeks	9 (1.0%)
Unknown duration	8
Previous TB	57 (6.2)
HIV status	
Tested negative	260 (28.3)
Tested positive	35 (3.8)
Self-report -Negative	199 (21.6)
Self report-Positive	103 (11.2)
Unknown status	322 (35.3)
CXR result	
Abnormal	464 (50.5)
Normal	401 (43.6)
Not done	54 (5.9)
Sputum results	
Sputum negative for TB	425 (46.2)
Sputum positive for TB	19 (2.1)
No sputum evaluated	475 (51.7)

## Results

Of 1067 bacteriologically confirmed index TB cases, consent to do contact tracing was obtained from 977 (91.6%), and 4297 individuals were enumerated from HH that were visited. Of the 4297 enumerated HH contacts, the majority of the household members were 15 years old and older; 2655 (61.8%) and children under 5 years were the least: 714 (16.6%) (Table 1.) The median number of people per household was 4 (IQR 3–6), the median number of rooms/ HH was 2 (IQR 2–3) and the median number of people per sleeping room/ HH was 2 (IQR 2–3).

Only 919 (21.4%) out of 4297 enumerated HH contacts presented to the health facility for screening during the study period (Table 2). The median age of HH contacts that presented was 15 (IQR 6–31), 549 (59.7%) were female and 663 (72.1%) did not have a current cough on presentation. HIV status was negative in 459 (49.9%), positive in 138 (15.0%) and unknown in 322 (35.0%). (Table 2).

**Fig. 2:**
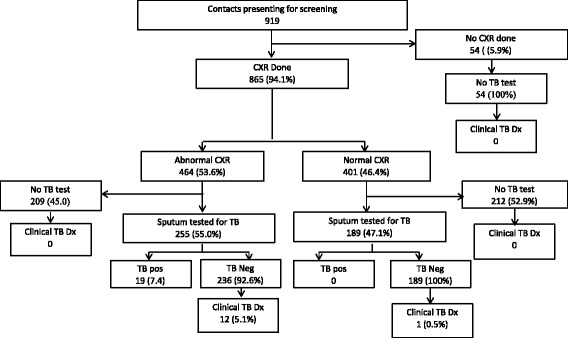
Diagnostic cascade of HH contacts

Of the 919 HH contacts that presented, 865 (94.1%) had a CXR done (Fig. 2). The CAD read 464/865 (53.6%) CXRs as abnormal. Of those with abnormal CXR, 255 submitted a sputum sample for evaluation and 19 patients had bacteriologically confirmed TB. Overall 444/919 (48.3%) of household contacts produced a sputum sample, including 444/865 (51%) of those who had a CXR. Among the 54 without a CXR, none produced a sputum sample. Characteristics of those that did not produce sputum are presented in Additional file 1: Table S1.

The total number of bacteriologically confirmed TB cases detected was 19 (4.3%) out of 444 who had a sputum sample evaluated representing 2.1% of all 919 who presented for screening. Overall, considering both bacteriologically confirmed and clinically diagnosed TB, 32 (7.2%) out 444 with evaluable sputum were diagnosed with TB, representing 3.5% of all 919 HH contacts that presented for TB screening.

**Fig. 3:**
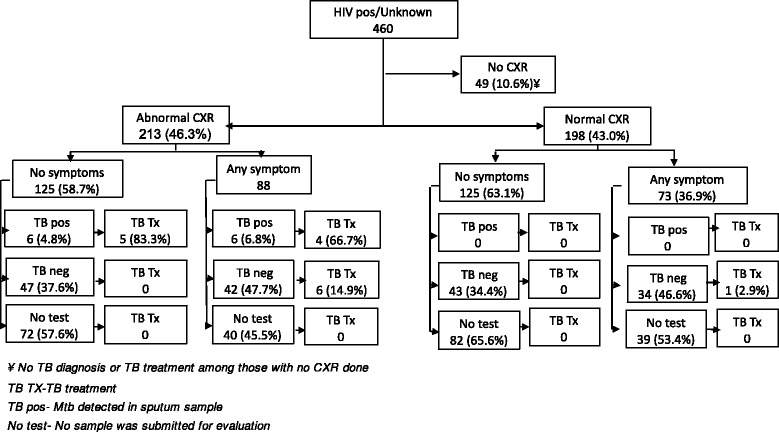
Diagnostic flow of HIV Positive/Unknown status HH contacts

Among 460 HIV positive/ status unknown, 411 had CXR done and 213 (51.8%) had a CXR read as abnormal. Of these with abnormal CXR, 125 (58.7%) had no symptoms of TB whilst 88 (41.3%) reported any symptom of TB (Fig. 3). Among 53 patients with no symptoms but with an abnormal CXR who submitted a sputum sample, TB was detected in six patients (11.3%). Among those with any symptom, who submitted a sputum sample, TB was detected in 6 (12.5%) out of 48 patients.

**Fig. 4:**
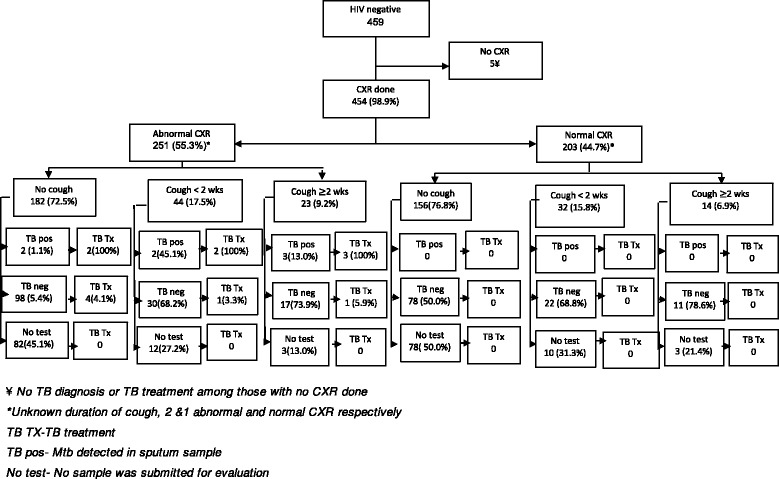
Diagnostic cascade of HIV negative HH contacts

Among 459 HIV negative HH contacts, 454 underwent CXR screening, 251 (55.3%) had CXR scored as abnormal and among those with abnormal CXR, 182 (72.5%) reported no cough, 44 (17.5%) reported a cough less than 2 weeks and 23 (9.2%) reported a cough of duration 2 weeks or more with or without other symptoms of TB (Fig. 4). Two HH contacts had unknown cough duration. Of those without a cough submitting sputum, 2 (2.0%) out 100 had TB detected. TB was detected in 2 (6.3%) out of 32 with cough less than 2 weeks and in 3 (15%) out of 20 with cough of 2 weeks or more. No TB was detected among those with a normal CXR.

Among HIV positive/ unknown status group; TB was clinically diagnosed in 6 (14.9%) out of 42 with any symptom. There was one clinical diagnosis of TB among those with any symptom but with a normal CXR. Among HIV negative HH contacts, TB was clinically diagnosed in 6 (4.1%) out of 145 HH contacts in whom sputum examination was negative but had an abnormal CXR. Details of clinical diagnosis in Additional file 2.

## Discussion

The traditional definition of presumptive TB that uses symptoms misses cases of TB [7, 8]. In this study, using digital CXR with CAD to define presumptive TB found active TB among symptom screen negative CXR abnormal HH contacts accounting for 42% (8 out of 19) of all bacteriologically confirmed TB cases detected. This finding suggests that CXR may have an important role in detection of TB especially among household contacts that may present early without typical symptoms of TB and active TB may be missed if dependent on symptom presentation [7–9]. Larger studies are required to ascertain the benefit of CXR screening in settings with high prevalence of TB and HIV where early detection and treatment are one of the TB control strategies.

Relying on symptom screen alone to define presumptive TB among PLHIV may be contributing to delayed diagnosis of TB, poor outcomes of HIV treatment and delayed IPT in patients that are wrongly designated as presumptive TB due to the low negative predictive value of symptom screen for TB [8, 21, 22]. The current WHO guidelines for IPT in resource constrained high TB and HIV burden settings recommend symptom screen to determine eligibility for IPT [5]. The Zambian guidelines for the management of TB in HIV infected persons recognises this limitation and recommends that all HIV positive individuals enrolling in care should be screened for TB using Xpert MTB RIF regardless of symptoms [23]. The sustainability of this strategy is questionable in view of the high number needed to screen to detect each case of TB and the high consumable costs of Xpert. Using pre-screening tools such as CXR can reduce the number needed to test for each case of TB that is detected and has the potential to reduce the cost of TB diagnosis [15, 16]. Careful costing studies are required to determine the costs and cost-effectiveness of using CXR as a pre-screening tool taking into consideration initial capital investments for CXR instruments and recurrent costs.

Availability of CXR in this study also meant that CXR was on hand for clinical evaluation of all those with a negative sputum result. It has already been demonstrated that availability of CXR at this primary care facility resulted in empirical treatment being started on the same day of presentation to the facility [19, 24], a significant finding given that diagnosis in sputum negative patients can take several weeks as patients are worked through the diagnostic algorithm for bacteriologically unconfirmed presumptive TB patients [25, 26]. Often CXR is not available at primary care facilities due the scarcity of qualified staff to operate and interpret CXRs. Digital CXR with CAD bypasses this barrier.

A low proportion of HH contacts presented for screening at the health facility. HH contact tracing plays a dual role; finding cases of TB as well as raising awareness of the risk of TB following close contact with a TB case. In this study, combined with HH contact tracing, HH members were given education on the relationship between TB and HIV, and encouraged to present to the facility for screening. The number of contacts that presented for screening could only be ascertained during the study period, once the study ended, it is not known how many may have accessed the health service as a result of the contact with HH contact tracing teams.

The limitation of this study is that a low proportion of enumerated HH contacts presented for screening. It might be that those that presented could be the ones with high risk for TB and may explain the observed high prevalence of TB in this population. Though this study does not provide accuracy of CXR compared to symptom screening, the findings in this study indicate and re-emphasise the need for systematic screening of high risk populations such as close contacts of TB patients for early detection of TB and provision of IPT.

## Conclusion

Despite the noted limitation of small sample size, this study adds evidence to the limitation of relying on symptom screen alone to identify presumptive TB patients for further testing with definitive TB tests. Symptom screen alone would have missed eight out 19 (42%) confirmed TB cases that were detected among HH contacts who had no symptoms of TB but had an abnormal CXR according to CAD. Symptom screen potentially misses early TB and might be contributing to the observed poor outcomes of HIV patients due to missed TB. There is need to consider use of other screening strategies such as CXR with CAD which has been observed to have a high sensitivity and high negative predictive value for TB to identify those at highest risk for TB who should be tested with follow on definitive TB testing. Larger evaluation studies of CAD are required that should include cost-effectiveness.

## Additional files

Additional file 1: Comparison of HH contacts that submitted sputum and those not submitting sputum. Contains comparison data of HH contacts that submitted sputum and those that did not submit sputum. (DOCX 18 kb)

Additional file 2: Clinically diagnosed patients with abnormal chest-xray. This file contains details of patients that were clinical diagnosed with TB based on CXR abnormality. It has time to TB diagnosis, comments on CXR abnormality that was observed, symptom screen and TB treatment outcome where available. (XLSX 31 kb)

## Abbreviations

CAD: Computer aided diagnosis
CXR: Chest x-ray
HH: Household
IPT: Isoniazid preventive therapy
PLHIV: People Living with HIV
SQL: Microsoft structured query language
TB: Tuberculosis
WHO: World Health Organisation
