# Supplementary material for: Digital CXR with computer aided diagnosis versus symptom screen to define presumptive tuberculosis among household contacts and impact on tuberculosis diagnosis
Source: BMC Infect Dis. 2017 Apr 24;17:301. doi: 10.1186/s12879-017-2388-7 (PMC5402643; doi:10.1186/s12879-017-2388-7)
Supplement: Supplementary file 1 — Comparison of HH contacts that submitted sputum and those not submitting sputum. Contains comparison data of HH contacts that submitted sputum and those that did not submit sputum. (DOCX 18 kb) [file 12879_2017_2388_MOESM1_ESM.docx]

| Characteristic | Total  N=919 | Sputum submitted  N=444 (48.3%)* | No Sputum submitted  N=475 (51.7%) * |
| --- | --- | --- | --- |
| **Age Group** |  |  |  |
| <=14 | 441(48.0%) | 95 (21.4%) | 346 (72.8%) |
| 15-24 | 154 (16.8%) | 110 (24.8%) | 44 (9.3%) |
| >=25 | 320 (34.8%) | 237 (53.4%) | 83 (17.5%) |
| Unknown age | 4 (0.4%) | 2 (0.4%) | 2 (0.5) |
| **Gender** |  |  |  |
| Female | 549 (59.7%) | 276 (62.2%) | 273 (57.5%) |
| Male | 370 (40.3) | 168 (37.8) | 202 (42.5%) |
| **CXR** |  |  |  |
| Abnormal | 464 (50.5%) | 255 (57.4%) | 209 (44.0%) |
| Normal | 401 (43.6%) | 189 (42.6%) | 212 (44.6) |
| Not done | 54 (5.9%) | 0 | 54 (11.4%) |
| **Previous TB** |  |  |  |
| No | 862 (93.8%) | 405 (91.2) | 457 (96.2) |
| Yes | 57 (6.2%) | 39 (8.85) | 18 (3.8%) |
| **Cough Duration** |  |  |  |
| No cough | 663 (72.1%) | 284 (64.0%) | 379 (79.8%) |
| Cough<2 weeks | 172 (18.7%) | 99 (22.3%) | 73 (15.4%) |
| Cough >= 2 weeks | 76 (8.3%) | 56 (12.6%) | 20 (4.2%) |
| Unknown duration | 8 (0.8%) | 5 (1.1%) | 3 (0.6%) |
|  |  |  |  |

Table S1: Comparison of HH contacts that submitted sputum and this not submitting sputum
